# Transcatheter stent implantation for long-segment coarctation in a young child: technical considerations and vascular safety using a large-diameter covered stent a case report

**DOI:** 10.1186/s12872-026-05907-5

**Published:** 2026-04-23

**Authors:** Vibhawari Pal, Aditya Shrivastav

**Affiliations:** 1 School of Allied Health Sciences, Datta Meghe Institute of Higher Education and Research, Sawangi, Wardha, Maharashtra 442001 India; 2 Swami Vivekananda Medical Mission’s Hospital, Khapri, Nagpur, Maharashtra 441108 India

**Keywords:** Coarctation of aorta, Paediatric cardiology, Covered stent, Transcatheter intervention, Vascular access, Case report

## Abstract

**Background:**

Severe long-segment coarctation of the aorta in children is traditionally managed surgically; however, transcatheter stent implantation is emerging as a viable alternative in selected cases. The use of large-diameter covered stents in small children raises concerns regarding vascular access, procedural safety, and future reintervention.

**Case presentation:**

An 8-year-old male child (21.7 kg) with global developmental delay presented with breathlessness. Clinical examination revealed radio-femoral delay, absent lower limb pulses, and upper limb hypertension (130/80 mmHg). Echocardiography demonstrated severe coarctation with left ventricular hypertrophy. CT aortography confirmed long-segment narrowing involving the aortic isthmus and proximal descending thoracic aorta, with a minimum diameter of 2.2 mm and a proximal descending aortic diameter of 12.3 mm. Cardiac catheterization showed an 80 mmHg peak systolic gradient. A transcatheter approach was undertaken. Right femoral arterial access was obtained using stepwise dilatation to accommodate a 12 Fr sheath. After systemic anticoagulation, a Zephyr XL covered stent (ZPXL 34) was deployed across the lesion under fluoroscopic guidance. Controlled expansion was performed, and mild residual narrowing was intentionally accepted to reduce the risk of aortic injury and allow staged dilatation. The post-procedural gradient decreased to 15 mmHg. Angiography confirmed satisfactory stent position with preserved left subclavian artery flow, and Doppler assessment showed no vascular complications. Lower limb pulses improved immediately.

**Conclusion:**

Transcatheter implantation of a large-diameter covered stent can be performed safely in selected young children with long-segment coarctation when meticulous attention is given to vascular access, device selection, and controlled, staged dilatation strategies.

**Supplementary Information:**

The online version contains supplementary material available at 10.1186/s12872-026-05907-5.

## Background

Coarctation of the aorta accounts for approximately 5–8% of congenital heart defects and is an important cause of childhood hypertension and cardiovascular morbidity [1, 2]. It is characterised by narrowing of the aortic lumen, most commonly at the juxtaductal region, resulting in upper body hypertension and reduced distal perfusion [3]. Long-segment coarctation, typically defined as narrowing extending beyond 10 mm, presents additional challenges due to lesion length, complex haemodynamics, and difficulties in achieving durable correction [4].

While surgical repair has traditionally been the standard treatment in younger children, transcatheter approaches have increasingly been used in selected cases. However, their application in small paediatric patients remains limited. The use of large-diameter covered stents in children under 10 years raises important concerns regarding vascular access, sheath-to-vessel mismatch, risk of vascular injury, and the need for future reintervention to accommodate somatic growth [5].

As a result, there is limited practical guidance on the safe execution of large-device transcatheter interventions in small children, particularly in the setting of long-segment disease. This report focuses on the technical considerations and vascular safety strategies involved in deploying a large-diameter covered stent in a young child, with emphasis on procedural planning, controlled expansion, and staged management.

## Case presentation

An 8-year-old male child (21.7 kg) with global developmental delay presented with breathlessness and previously diagnosed severe coarctation of the aorta. Developmental delay had been identified in early infancy, with ongoing pharmacological management for behavioural symptoms (Table 1).

**Table 1:** Baseline clinical characteristics

Parameter	Findings
Age	8 years
Sex	Male
Weight	21.7 kg
Presenting symptom	Breathlessness
Relevant comorbidity	Global developmental delay
Upper limb blood pressure	130/80 mmHg
Lower limb pulses	Absent (dorsalis pedis, posterior tibial)
Radio-femoral delay	Present
Cardiac auscultation	Ejection systolic murmur (left infraclavicular, radiating to back)
Left ventricular function	Preserved systolic function

On cardiovascular examination, radio-femoral delay was present, with palpable brachial and radial pulses and absent dorsalis pedis and posterior tibial pulses bilaterally. Right upper limb blood pressure was 130/80 mmHg. Cardiac auscultation revealed an ejection systolic murmur over the left infraclavicular region radiating to the back (Table 2).

**Table 2:** Multimodality imaging findings

Imaging Modality	Key Findings
Transthoracic echocardiography	Severe long-segment coarctation, turbulent flow, LV hypertrophy, preserved LV systolic function
CT aortography	Long-segment narrowing involving isthmus and proximal descending aorta
Narrowest aortic diameter	2.2 mm
Proximal aortic diameter	12.3 mm
Invasive aortography	Severe long-segment narrowing with post-stenotic dilatation
Peak systolic gradient (catheterization)	80 mmHg

**Fig. 1:**
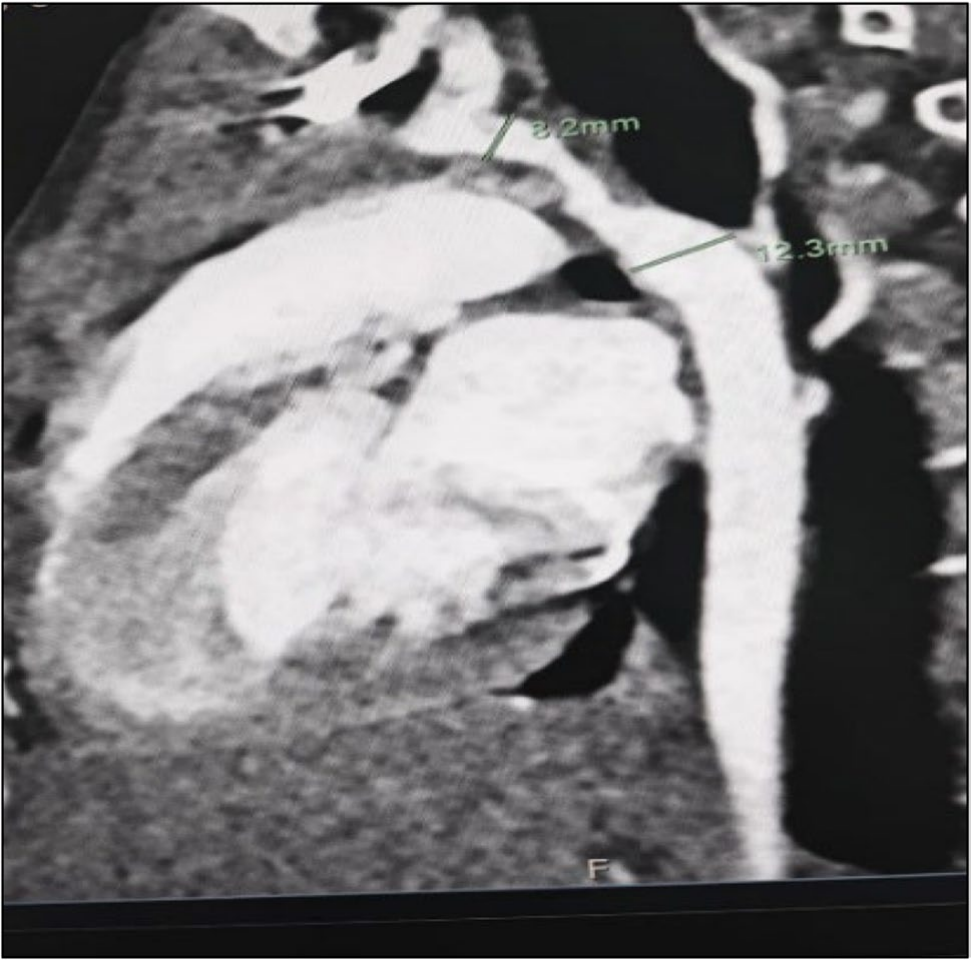
CT aortography (sagittal view) demonstrating severe long-segment coarctation of the aorta. The narrowest segment measures 2.2 mm (upper measurement), with proximal aortic diameter of 12.3 mm (lower measurement), showing marked discrepancy between pre-stenotic and stenotic segments

**Fig. 2:**
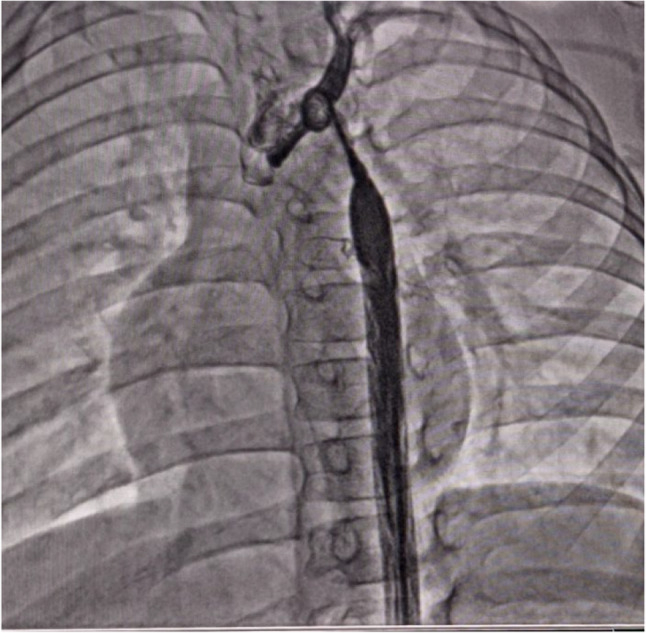
Pre-intervention aortogram showing severe long-segment coarctation with marked narrowing of the aortic isthmus and proximal descending thoracic aorta

Transthoracic echocardiography demonstrated severe long-segment coarctation with preserved left ventricular systolic function and features of pressure overload. Contrast-enhanced CT aortography confirmed long-segment narrowing involving the aortic isthmus and proximal descending thoracic aorta, with a minimum luminal diameter of 2.2 mm and a proximal descending aortic diameter of 12.3 mm (Fig. 1). There were no imaging or laboratory features suggestive of inflammatory aortitis. Invasive haemodynamic assessment during cardiac catheterisation demonstrated a peak systolic gradient of 80 mmHg across the coarctation segment. Pre-intervention angiography confirmed severe long-segment narrowing with post-stenotic dilatation (Fig. 2).

Following multidisciplinary discussion, a transcatheter approach was selected based on anatomical suitability and clinical considerations, including the patient’s neurodevelopmental status. A key diagnostic and therapeutic challenge in this case was determining the suitability of transcatheter intervention over surgical repair in a young child with long-segment coarctation, given concerns regarding vascular access limitations, large sheath requirements, and risk of aortic wall injury (Table 3).

**Table 3:** Haemodynamic parameters assessed before and after intervention

Parameter	Pre-intervention	Post-intervention
Peak systolic gradient across coarctation	80 mmHg	15 mmHg
Upper limb blood pressure	130/80 mmHg	110/70 mmHg
Lower limb pulses	Absent	Palpable
Echocardiographic flow pattern	Turbulent	Laminar
Left ventricular systolic function	Preserved	Preserved

The procedure was performed under local anaesthesia with conscious sedation. Right femoral arterial access was obtained using a landmark-guided technique, followed by stepwise dilatation to accommodate a 12 Fr sheath. Intravenous unfractionated heparin (100 IU/kg) was administered following arterial access, with additional dosing guided by activated clotting time (ACT) monitoring.

A 0.035″ Amplatz Super Stiff guidewire (Boston Scientific) was advanced across the lesion and positioned in the ascending aorta to provide stable support for device delivery. Angiographic assessment confirmed that the coarctation segment was distal to the left subclavian artery with an adequate proximal landing zone (approximately 8–10 mm from the left subclavian artery).

A Zephyr XL covered stent (ZPXL 34) was selected based on lesion length and reference vessel diameter. The stent was mounted on a Balloon-in-Balloon (BIB) catheter (NuMED), sized approximately 12 mm × 40 mm to match the proximal descending aortic diameter. Deployment was performed under fluoroscopic guidance using a controlled, staged expansion technique. Full expansion was intentionally avoided, and mild residual narrowing was accepted to reduce the risk of aortic wall injury and to allow for future redilatation. This strategy reflects a planned staged therapeutic approach, allowing gradual vessel adaptation and reducing procedural risk while preserving the option for future redilatation.

**Fig. 3:**
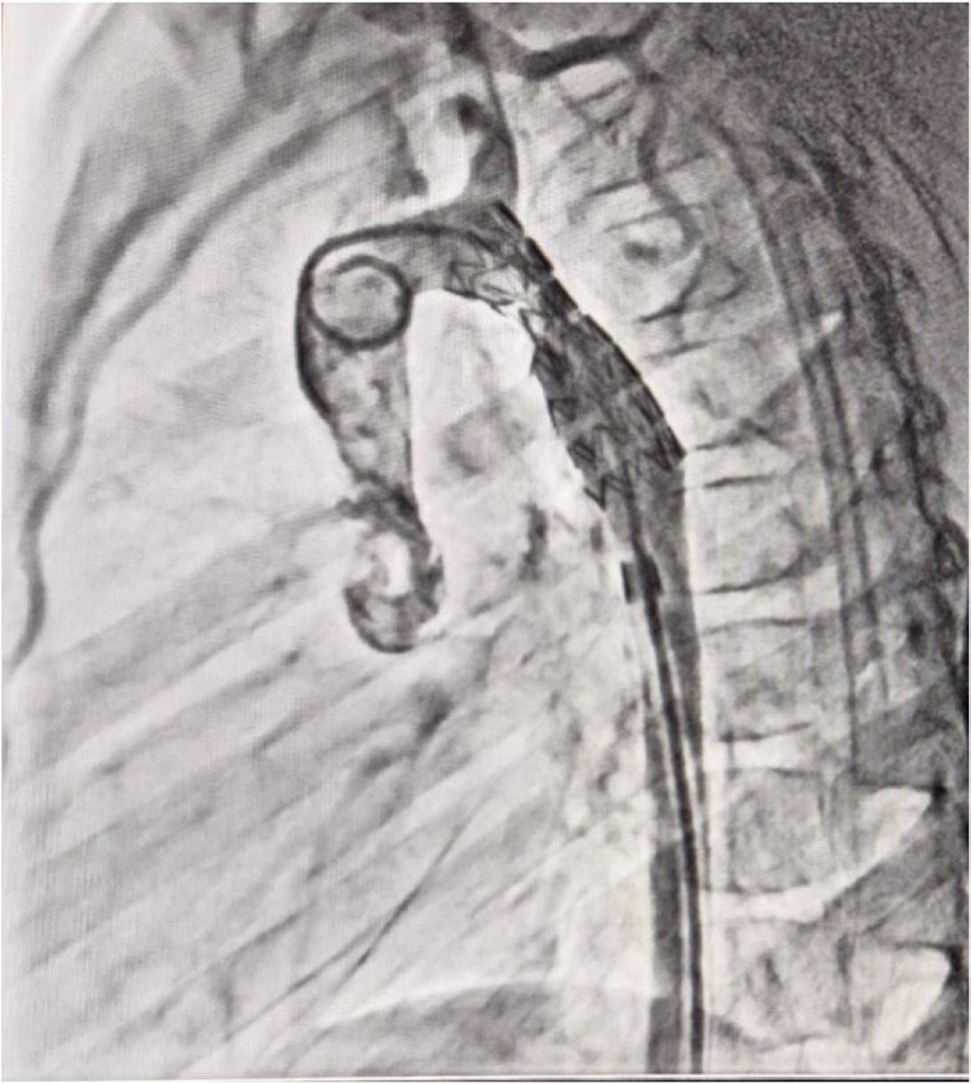
Post-intervention aortogram demonstrating deployment of a Zephyr XL covered stent (ZPXL 34) with restoration of normal aortic calibre

Post-deployment angiography demonstrated satisfactory stent position with restoration of aortic calibre, no evidence of dissection or perforation, and preserved flow in the left subclavian artery (Fig. 3). The invasive peak systolic gradient decreased from 80 mmHg to 15 mmHg. Immediate echocardiography confirmed abolition of the arch gradient with restoration of laminar flow. Lower limb pulses became palpable following the procedure.

The post-procedural course was uneventful. The patient was discharged on antiplatelet therapy, with improvement in blood pressure (110/70 mmHg) and no requirement for antihypertensive medication. No vascular access complications, stent-related adverse events, or early restenosis were observed. Follow-up evaluation showed stable haemodynamics and no evidence of early restenosis. Long-term surveillance with clinical and imaging follow-up is planned, with consideration of future stent dilatation in accordance with somatic growth. The patient remained compliant with prescribed antiplatelet therapy and scheduled follow-up evaluations.

### Patient perspective

The patient’s caregivers reported noticeable improvement in activity levels and reduction in breathlessness following the procedure. They expressed satisfaction with the minimally invasive approach and the early recovery, particularly given the child’s underlying neurodevelopmental condition.

## Discussion

Long-segment coarctation of the aorta in young children presents unique technical challenges that extend beyond those encountered in discrete lesions. The combination of severe narrowing, extended lesion length, and small vessel calibre increases the risk of procedural complications, particularly when large delivery systems are required [4].

In this case, transcatheter stent implantation was selected as an alternative to surgical repair based on anatomical suitability and clinical considerations. In children above a certain weight threshold, transcatheter therapy has increasingly become an accepted approach, demonstrating favourable outcomes in appropriately selected patients [4]. However, the use of a large-diameter covered stent in a child weighing 21.7 kg required careful attention to vascular access and procedural strategy.

Introduction of a large sheath in a small femoral artery carries a recognised risk of vascular injury. This risk was mitigated by a stepwise dilatation approach, allowing gradual accommodation of the sheath and reducing access-related complications. Careful procedural technique is essential in paediatric interventions, where vessel size and access limitations remain a major concern.

Device selection was guided by lesion characteristics. The long-segment and severely narrowed anatomy increased the risk of aortic wall injury during rapid expansion. Covered stents are known to reduce the risk of complications such as dissection and aneurysm formation by providing structural support to the vessel wall and limiting uncontrolled expansion [2]. Additionally, the selected stent platform allows for future redilatation, which is an important consideration in paediatric patients to accommodate somatic growth.

A key procedural decision was the intentional avoidance of full expansion during initial deployment. Excessive balloon dilatation in tight coarctation may predispose to complications such as dissection, rupture, or aneurysm formation [2, 5]. A staged approach with controlled expansion allows gradual vessel adaptation while achieving meaningful haemodynamic improvement. This strategy has been supported in interventional practice, where avoiding aggressive dilation reduces procedural risk and improves safety outcomes [5, 7].

The anatomical relationship of the coarctation segment to the left subclavian artery was carefully assessed to ensure an adequate landing zone and to avoid compromise of branch vessel flow. Accurate anatomical assessment is essential for procedural planning and safe device deployment in complex coarctation [1, 6, 8]. 

Patient-specific factors also influenced management. The presence of neurodevelopmental delay influenced procedural planning, particularly favouring conscious sedation to allow early neurological assessment and avoid potential challenges associated with general anaesthesia in a behaviourally vulnerable child. Individualised management strategies are particularly important in paediatric populations with associated comorbidities, including anticipated challenges in post-procedural compliance and follow-up.

Although imaging played an important role in anatomical assessment, the primary contribution of this case lies in demonstrating the feasibility and safety of large-sheath, covered stent implantation in a small child when meticulous procedural planning and technique are employed.

## Limitations

This report describes a single case, limiting generalisability. Follow-up duration is short, precluding assessment of long-term outcomes, including stent durability, vessel growth adaptation, and the need for repeat intervention. Long-term surveillance with serial clinical and imaging follow-up is essential to evaluate late complications and guide future redilatation strategies [1, 9, 10] .

## Conclusion

This case demonstrates that transcatheter stent implantation using a large-diameter covered stent can be performed safely in selected young children with long-segment coarctation when meticulous attention is given to vascular access, device selection, and controlled, staged dilatation. It highlights the importance of procedural planning and technical precision in minimising complications and optimising outcomes in high-risk paediatric interventions.

## Supplementary Information

Supplementary Material 1.

## Data Availability

All data generated or analysed during this study are included in this published article. Additional clinical data are available from the corresponding author upon reasonable request, subject to patient confidentiality and institutional data-sharing policies.

## Abbreviations

BP: Blood pressure
CHD: Congenital heart disease
CoA: Coarctation of the aorta
CT: Computed tomography
LV: Left ventricle
LVH: Left ventricular hypertrophy
mmHg: millimetres of mercury
ZPXL: Zephyr XL covered stent platform
