# Supplementary material for: Transcatheter stent implantation for long-segment coarctation in a young child: technical considerations and vascular safety using a large-diameter covered stent a case report
Source: BMC Cardiovasc Disord. 2026 Apr 23;26:484. doi: 10.1186/s12872-026-05907-5 (PMC13251018; doi:10.1186/s12872-026-05907-5)
Supplement: Supplementary file 1 — Supplementary Material 1. [file 12872_2026_5907_MOESM1_ESM.pdf]

CARE Checklist of information to include when writing a case report

CARE case report guidelines

CC BY-NC-ND

Topic	Item	Checklist item description	Reported on Line
**Title**	1	The diagnosis or intervention of primary focus followed by the words "case report"	Page 1, Lines 1–3
**Key Words**	2	2 to 5 key words that identify diagnoses or interventions in this case report, including "case report"	Page 2, Lines 60–63
**Abstract (no references)**	3a	Introduction: What is unique about this case and what does it add to the scientific literature?	Page 2, Lines 32–36
	3b	Main symptoms and/or important clinical findings	Page 2, Lines 37–45
	3c	The main diagnoses, therapeutic interventions, and outcomes	Page 2, Lines 45–56
	3d	Conclusion—What is the main "take-away" lesson(s) from this case?	Page 2, Lines 57–61
**Introduction**	4	One or two paragraphs summarizing why this case is unique (**may include** references)	Page 2–3, Lines 63–79
**Patient Information**	5a	De-identified patient specific information	Page 3, Lines 80–82
	5b	Primary concerns and symptoms of the patient	Page 3, Lines 80–83
	5c	Medical, family, and psycho-social history including relevant genetic information	Page 3, Lines 80–85
	5d	Relevant past interventions with outcomes	Page 3, Lines 82–85
**Clinical Findings**	6	Describe significant physical examination (PE) and important clinical findings	Page 3, Lines 83–86
**Timeline**	7	Historical and current information from this episode of care organized as a timeline	Page 10–12, Tables 1–3
**Diagnostic Assessment**	8a	Diagnostic testing (such as PE, laboratory testing, imaging, surveys).	Page 3, Lines 86–93
	8b	Diagnostic challenges (such as access to testing, financial, or cultural)	Page 3, Lines 93–100
	8c	Diagnosis (including other diagnoses considered)	Page 3, Lines 90–93
	8d	Prognosis (such as staging in oncology) where applicable	Page 4, Lines 123–130
**Therapeutic Intervention**	9a	Types of therapeutic intervention (such as pharmacologic, surgical, preventive, self-care)	Page 3–4, Lines 100–118
	9b	Administration of therapeutic intervention (such as dosage, strength, duration)	Page 3–4, Lines 100–110
	9c	Changes in therapeutic intervention (with rationale)	Page 4, Lines 110–118
**Follow-up and Outcomes**	10a	Clinician and patient-assessed outcomes (if available)	Page 4, Lines 120–126
	10b	Important follow-up diagnostic and other test results	Page 4
	10c	Intervention adherence and tolerability (How was this assessed?)	Page 4, Lines 126–128
	10d	Adverse and unanticipated events	Page 4, Lines 120–122
**Discussion**	11a	A scientific discussion of the strengths AND limitations associated with this case report	Page 5–6, Lines 147–184
	11b	Discussion of the relevant medical literature **with references**	Page 4–5, Lines 125–147
	11c	The scientific rationale for any conclusions (including assessment of possible causes)	Page 4–5
	11d	The primary "take-away" lessons of this case report (without references) in a one paragraph conclusion	Page 6, Lines 185–193
**Patient Perspective**	12	The patient should share their perspective in one to two paragraphs on the treatment(s) they received	Page 4, Lines 128–132
**Informed Consent**	13	Did the patient give informed consent? Please provide if requested	Yes ☒ No ☐
